# Cell secretion and membrane fusion: highly significant phenomena in the life of a cell

**DOI:** 10.15190/d.2014.22

**Published:** 2014-09-18

**Authors:** Mircea Leabu, Garth L. Nicolson

**Affiliations:** University of Medicine and Pharmacy "Carol Davila", and "Victor Babes" National Institute of Pathology, Bucharest, Romania; Department of Molecular Pathology, Institute for Molecular Medicine, Huntington Beach, California, 92647 USA

**Keywords:** cell secretion, membrane fusion, porosome, exosomes, electron microscopy, cancer, mathematical approach, secretory vesicle, science history

## Introduction

Is there any cell that does not secrete something necessary for maintenance of the organism? Secretion involves membrane fusion, which is important in assuring good intra- and extra-cellular organization, transport of essential nutrients, synthesis and delivery of vital cellular components, destruction and removal of damaged cellular materials and ultimately survival of every cell. Cells are compartmentalized by various membrane structures into organelles that are responsible for biosynthesis, energy production, replication, transportation, recycling as well as secretion, and membrane fusion events are required to form and replace these structures. Thus membrane fusion along with membrane trafficking are critical to every cell’s existence, and they must be very well coordinated and controlled. Membrane trafficking, which involves vesicular budding of the source membrane, directed transport and eventually fusion with the target membrane is a very specific process. All of these processes depend, in particular, on basic principals of biological membrane structure and dynamics, a topic that was reviewed recently in this journal^1^.

## Vesicle Trafficking Inside Cells

In 2013 The Nobel Prize for medicine or physiology was jointly awarded to James E. Rothman, Randy W. Schekman and Thomas C. Südhof, "for their discoveries of machinery regulating vesicle traffic, a major transport system in our cells"^2^. However, despite the impressive deepening of our knowledge about cell secretion and membrane fusion, there are several gaps that have to be filled and some controversies that need to be addressed.

This special issue is a modest attempt to advance in a small way the field of cell secretion and membrane fusion. Usually, a goal of every Special Issue in a scientific journal is to present state-of-the-art knowledge in the field or parts of a field and to launch challenges for the future work, but they can also offer opportunities for clarifying terms and/or concepts. Sometimes, these opportunities are neglected. In this Special Issue, we intended to use such an opportunity.

## Cell Secretion and Membrane Fusion

Cell secretion is a very useful and practical process for integrative organization and functioning of cells. For example, the very elaborate cellular trafficking pathway, cell secretion, needs coherent cooperation between various organelles and other complex cellular structures that control molecular biosynthesis, processing and storage as cargos in secretory vesicles of a plethora of macromolecules destined for exocytosis. The passage of the secretory molecules form one membrane-bound organelle to another (from endoplasmic reticulum – ER to Golgi apparatus) and further on to secretory vesicles cannot be elaborated without necessary membrane fissions and fusions, while the final event, exocytosis, also requires membrane fusion.

It usually takes scientists a long time to advance knowledge, in this case about cell secretion, from intuition to proof. The first significant transition in our understanding about cell secretion was made in 1964 by Caro and Palade^3^, who reported that proteins to be secreted by pancreatic acinar cells are biosynthesized at the ER level, pass throughout the Golgi complex and then to secretory vesicles to be released through the apical membrane inside the lumen of the acinus. Nothing about the molecular mechanisms during the described steps in this secretory pathway was known at the time.

The notion that membrane fusion is related to cell secretion was mentioned for the first time almost a decade later, in 1973^4^, and about that same time (1971) a theory on membrane fusion was advanced^5^ that described at least four stages in the process: “membrane contact; induction; fusion; and stabilization”. However, details on the molecular mechanisms that characterize membrane fusion were significantly deciphered only in the last decade of the twentieth century, when specific membrane proteins were described that orchestrated with exquisite precision the fusion of the correct membranes at the right moment and location (see reference^6^ as one of the first reviews).

## The Complexity of Secretion

Data have been accumulating that demonstrate the complexity of cell secretion pathways and the individual sequential events along their pathways. Every event in the adventure of a secretory (macro)molecule: biosynthesis and processing, transport through different organelles and their arrival at secretory vesicles and eventually at the plasma membrane, with fissions and fusions of the corresponding membranes, indicates diversity of molecular and cellular phenomena. For example, different types of exocytosis were previously described in terms of the mechanisms involved in each step of the process and the cooperation of secretory vesicles and target membranes. Terms were developed for such phenomenon, such as “full fusion”, “kiss-and-run” or "kiss-and-coat”^7,8^. In addition, there were also two major categories of exocytosis that were described in terms of a cell’s decision to secrete materials: spontaneous or stimulated (alternatively constitutive or signaled).

With respect to these two classifications mentioned above, we have to point out an often improperly used term for the second category. Although in general it does not matter which pair of terms is preferred or used, many scientists use the term “regulated exocytosis” for stimulated/signaled exocytosis. We consider this term somewhat confusing, if not contradictory to our current understanding of cell organization and function. Our current knowledge suggests that most if not all events in cells are regulated events. Nothing is considered outside of a cell’s control, even if its behavior is modified by outside factors that are beyond its control. In the worst-case scenario, a cell can decide to commit to programmed cell death or apoptosis, and even this decision is a regulated one. Therefore, we consider it helpful and useful to advocate for the use of the term “signaled exocytosis,” or its alternative “stimulated exocytosis,” instead of “regulated exocytosis.” That is because both constitutive/spontaneous and signaled/stimulated exocytosis are subtly controlled and regulated by highly elaborate cellular mechanisms.

## Morphological Evidence of Secretory Vesicles

This Special Issue of Discoveries is titled “Cell Secretion and Membrane Fusion”, and it is focused on the last event of the secretory pathway, exocytosis, and the newly discovered machinery facilitating membrane fusion. It also considers the secreted materials’ discharge into a structure called the porosome. There are two original contributions and five reviews included in this special issue. It is worthwhile to mention that one of the reviews is a mathematical approach or description of cell secretion and another one deals with excretion of materials encased by membranes called exosomes.

Using electron microscopy in his original paper on secretion Lloyd L. Anderson^9^ investigated the morphological diversity of secretory vesicles (filled, empty and partially empty) in porcine pituitary gland, and he found variations in their ratios after stimulation with GH secretagogue, without any significant variations in the total vesicle number. Then using ultrastructural immunogold cytochemistry he showed the presence of growth hormone only in the electron dense, filled vesicles. The results corresponded to a “kiss-and-run” mechanism, whereby porosomes required specific properties for transient docking and membrane fusion during secretion.

## Stressogenic Factors and Secretion

We can define the microenvironment that, *inter alia*, induces secretion as a stressogenic one in terms of its (bio)chemical, (bio)physical and social characteristics. This stressogenic environment has an important impact on organisms, by affecting more or less (depending on the stress factor) the functions of different cells in various tissues. The effects of one stress factor, namely white noise, on the organization and function of neuronal porosomes in the cat were investigated in the original paper by Mzia G. Zhvania and her collaborators^10^ and discussed in the current Special Issue. Using transmission electron microscopy, the authors studied the effects of continuous white noise on porosome ultrastructural features (diameter and depth) in inferior colliculi and in the medial geniculate body neuronal synapses. They found significant variations in the numerical values characterizing the porosome depth in the neurons in the medial geniculate body and pointed out that these effects may reflect alterations in neurotransmission in the subcortical auditory area.

## Mathematical Models

Numbers, but also equations, are used as defining scientific elements in the paper presented by Ilan Hammel and Isaac Meilijson^11^. Biology definitely needs mathematical models for understanding and dealing with its phenomena in an anticipative manner (in both cellular physiology and pathology). The multiparametric system represented by a cell, where every parameter can vary in an either independent or controlled manner, requires a very special mathematical approach applicable to both cell ultrastructural elements and molecules within the cell. Beyond the numbers and equations, the biological significance of these approaches is important for the progress of our knowledge in cellular biology. The article applies mathematics to secretory granule biogenesis and maintenance, the porosome being an “agent” controlling them. To our understanding this paper represents a quantification of maintaining the right balance between order and disorder in cell organization and functioning and in the communication pathways assuring successful survival of cells (a balance that is critical for molecules up to complex structures). Parameters such as detailed organization (even in terms of chemical composition), size/geometry, interaction abilities, amounts of “carriers of communicating signals” (meaning all the biochemicals in a cell) determine the development of an event (initiation, intensity, rate, etc.) that acts as stimulus, inhibitor or noise. The mathematical approach describing complex biological phenomena, and the interpretation of the results developed by the authors in their article, could be applied to understanding other cellular processes, such as a decision for crinophagy, a particular form of autophagy in secretory cells.

## The Porosome

The contributions of Constantin Craciun^12^ have provided us with an arch over time linking the creativity and pioneering contributions of Professor George E. Palade, discoverer of the ribosome, and his student Bhanu Jena, discoverer of the porosome. A similar idea could be found in an Editorial published in Pancreatology^13^. Beyond this historical reminder, this review discusses findings at the ultrastructural level, by use of high quality electron micrographs, on the organization of the porosome in exocrine pancreas. The elements expressed on the cytosolic surface are able to tether the base of the porosome to the zymogen granule membrane. Moreover, Craciun’s work has proved that the same secretory vesicle can dock at more than one porosome.

Keeping with this pioneering discovery of the porosome and coining the name of the structure, Bhanu Jena has contributed to this Special Issue by providing a review related to the significance of porosome function in balancing cellular behavior between normal physiology and pathology^14^. This paper demonstrates the intimate relationships between the complex organization of a porosome and the impact of each protein on the function of this nano-structure. Using as an experimental cell model, the Calu-3 cell line, Jena reports cooperation between proteins organizing the porosome in airway epithelia that assures the optimal viscosity of the secreted mucus and its direction toward the exterior of the respiratory pathway. Otherwise, cystic fibrosis could be developed. As a leader in the field, his review opens a door toward future studies on the molecular pathology of respiratory diseases.

## Molecular Mechanisms and the Porosome

In their review, Mircea Leabu and Cristina Mariana Niculite point out the significance of porosome discovery for the field of cell secretion and towards deciphering the molecular mechanisms of secretion^15^. This review extends a mini-review, published in 2006, on cell secretion and membrane fusion, discussing the porosome soon after the term was coined^16^. There are two contributions that should be mentioned regarding this review, a historical one and a didactical one. For the historical direction in the paper, there is an interesting description of secretion, from its intuition to experimental proof. The authors have also prepared a table related to the chronology of the events in porosome discovery. We invite the readers to compare this information with the interesting information presented in the interview given by Dr. Bhanu Jena, the discoverer of the porosome^17^. In our opinion, this information is very useful for the education of young scientists, because we often forget or ignore the wise lessons of history. The didactical weight of this review is exemplified by the authors’ concern in defining the porosome, a nano-structure embedded in the plasmalemma, as a membrane microdomain, and trying to remove some putative confusion that considers it as an organelle. Definitely, elements of the organization of a membrane cannot be considered an organelle. Otherwise, lipid rafts, junctions, signalosomes, caveolae, coated pits/vesicles etc. would have to be considered as organelles. This could also be seen as a contribution clarifying the category of which the porosome belongs. Moreover, the porosome can be considered as one more discovery confirming the validity of the Fluid Mosaic membrane model^18^, in addition to other information accumulated over time as discussed in a recent review in this journal^19^.

## Exosomes or Extracellular Vesicles

Last, but not least, an interesting review in the field of cell secretion is the contribution of Dipak Datta’s research team^20^. This review emphasizes the diversity of extracellular vesicles, their complexity at the molecular level and the property that they are both released (by some cells) and taken up (by other cells), indicating a possible communication link between cells. Therefore, wondering why and for what purposes these extracellular vesicles are secreted makes sense, because they are not just eliminated by cells as a simple waste product. Moreover, the cell microenvironment cannot be considered a garbage bin. Beyond these subliminal questions, the review approaches issues related to exosomes (one type of extracellular vesicles) released by cancer cells, which, through their molecular components, regulate tumor growth, angiogenesis (critical for a tumor to develop), metastasis or drug resistance. All these issues justify the interest for studying exosomes in terms of their biogenesis and secretion, molecular characterization, the specificity of their uptake by target cells or their impact in cell physiology and pathology.

## Special Thanks to Our Contributors and Reviewers

The Guest Editors of this Special Issue wish to thank the authors and reviewers that contributed to its successful publication. It was not easy to find reviewers for some of these contributions, and this has stimulated us to specially acknowledge the efforts of those experts. All the involved authors, reviewers, and editors have tried to prepare an interesting and challenging Special Issue for Discoveries. We are conscious that many other interesting topics would have increased the impact of our attempt. By this editorial, we, the Guest Editors, have attempted to attract readers to this Special Issue by summarizing some of the interesting features of each published article.
